# National Consensus Catalog of Psychomotor Competencies for Brazilian Medical Graduates: a modified Delphi study – Part I

**DOI:** 10.1590/1806-9282.20261096

**Published:** 2026-10-19

**Authors:** Rodrigo de Melo Nogueira, José Eduardo Lutaif Dolci, Cesar Eduardo Fernandes, Antonio José Gonçalves, José Gustavo Parreira, Ramiro Colleoni, Francisco Sandro Menezes-Rodrigues, Fernando Sabia Tallo, Brazilian Specialty Societies

**Affiliations:** 1 Universidade Federal de São Paulo – São Paulo (SP), Brazil.; 2 Santa Casa de São Paulo, Faculdade de Ciências Médicas – São Paulo (SP), Brazil.; 3 Faculdade de Medicina do ABC – Santo André (SP), Brazil.

**Keywords:** Education, medical, Curriculum, Clinical competence, Delphi technique, Psychomotor performance, Competency-based medical education

## Abstract

**OBJECTIVE:**

To develop a national consensus catalog of essential psychomotor competencies and establish the minimum expected competence levels for graduating medical students across anesthesiology and surgical specialties in Brazil using a modified Delphi process.

**METHODS:**

This national consensus-development study used a two-round modified Delphi process coordinated by the Brazilian Medical Association. Scientific Committees representing anesthesiology and 14 surgical specialty societies developed preliminary specialty-specific catalogs and proposed the expected competence level for each competency. Board-certified specialists evaluated the competency–competence-level combinations using a five-point Likert scale. Consensus was predefined as ≥75% combined agreement.

**RESULTS:**

A total of 802 specialists participated in the first Delphi round, and 622 completed the second round. The preliminary catalogs comprised 343 competency statements. Of these, 273 (79.6%) achieved consensus, whereas 70 (20.4%) were excluded. During the first Delphi round, participants proposed 103 additional competency statements, of which 90 (87.4%) achieved consensus after evaluation during the second Delphi round. The final consensus catalog comprised 363 specialty-specific psychomotor competencies, corresponding to 244 unique psychomotor competencies after harmonization. Most competencies were classified as Perform independently (n=274), followed by Perform under supervision (n=55) and Master the technique (n=34).

**CONCLUSION:**

This modified Delphi study established a national consensus catalog of psychomotor competencies and expected competence levels for graduating medical students across anesthesiology and 14 surgical specialties. The catalogue provides a national consensus-based reference for competency-based undergraduate medical education. Future studies should evaluate its implementation and educational impact.

## INTRODUCTION

The primary goal of undergraduate medical education is to prepare graduates to provide safe, effective, and patient-centered care by acquiring essential psychomotor competencies before entering supervised clinical practice^1^. These competencies are fundamental to the safe performance of diagnostic and therapeutic procedures. However, substantial variability persists among medical schools regarding which competencies should be taught, assessed, and expected at graduation. This heterogeneity underscores the need for nationally standardized educational references to support curriculum development, procedural training, and learner assessment^2^.

Over the past two decades, competency-based medical education has transformed undergraduate training, while national frameworks and Delphi studies have defined expected competencies within specific specialties and educational contexts. Despite these advances, no nationally developed consensus catalogue has comprehensively defined essential psychomotor competencies across multiple surgical specialties and anesthesiology for undergraduate medical education. Existing competency frameworks primarily define broad educational outcomes rather than explicit psychomotor competencies, and published Delphi studies have focused on individual specialties or isolated procedures. Consequently, no nationally developed consensus catalogue harmonizes psychomotor competency expectations for undergraduate procedural education^3,4,5,6^.

In Brazil, the National Curriculum Guidelines define the general competencies expected of graduating physicians but do not establish a nationally standardized catalogue of psychomotor competencies. Consequently, medical schools independently define procedural training, competency assessment, and performance standards. To address this gap, Scientific Committees from the Brazilian Medical Association (*Associação Médica Brasileira* – AMB)-affiliated specialty societies developed preliminary psychomotor competency catalogues that subsequently underwent national consensus through a structured modified Delphi process^7,8,9^. This study aimed to develop a national consensus catalogue of essential psychomotor competencies and establish minimum expected competence levels for Brazilian medical graduates across surgical specialties and anesthesiology using a modified Delphi process. The catalogue provides a standardized framework for curriculum development, simulation-based training, competency assessment, faculty development, and educational policy^10^.

## METHODS

### Study design

This national consensus-development study used a two-phase modified Delphi design to develop a national catalogue of essential psychomotor competencies and establish the minimum expected competence level for graduating medical students across anesthesiology and surgical specialties in Brazil. The study was coordinated by the Brazilian Medical Association and conducted according to DELPHISTAR and ACCORD recommendations for Delphi studies^10,11,12,13,14,15^. The overall study design is presented in Figure 1.

**Figure 1:**
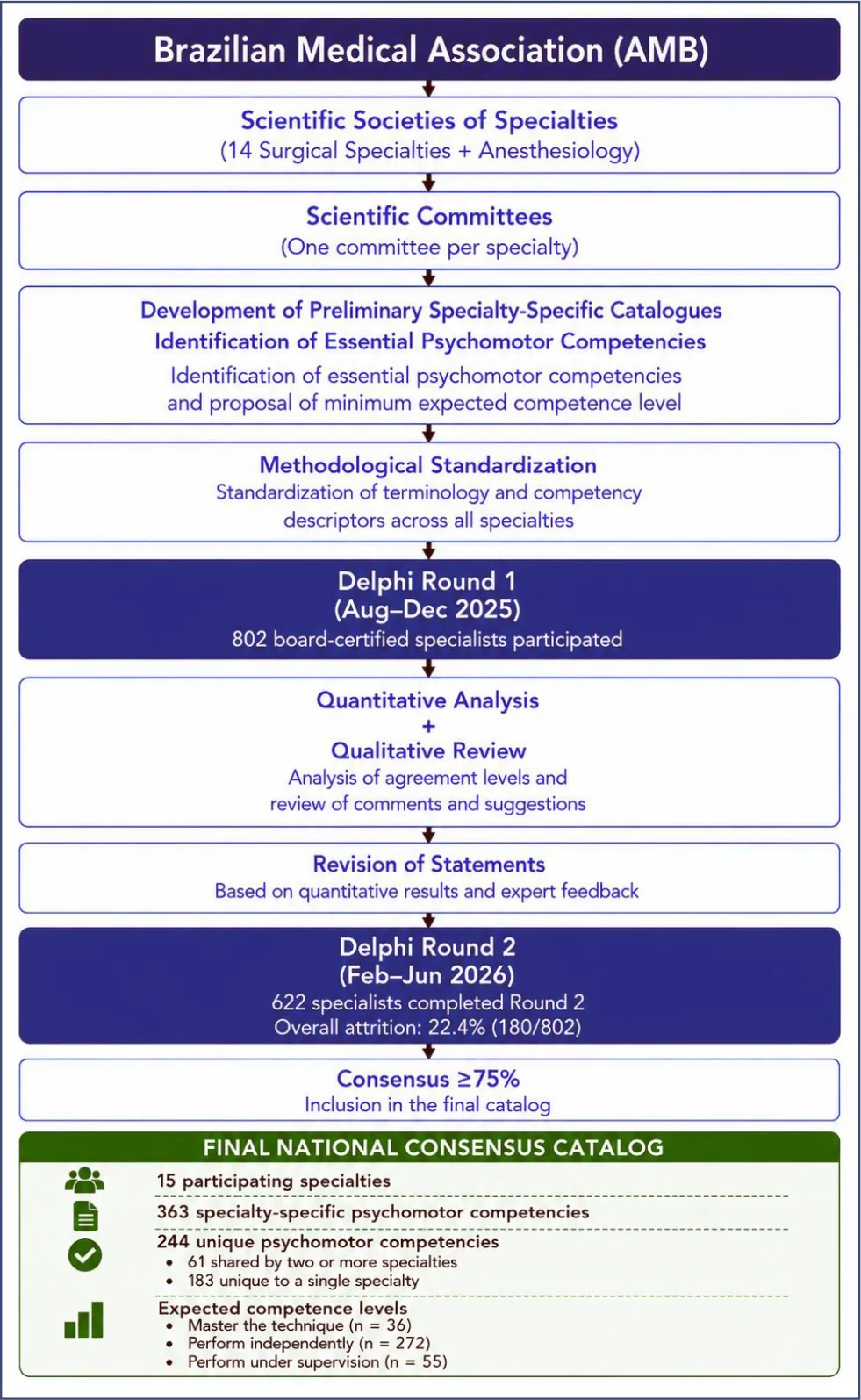
Study desing and modified Delphi consensus process for the development and national validation of the Brazilian catalogue of essential psychomotor competencies.

### Phase 1: Development of preliminary catalogues

Phase 1 was conducted between February and June 2025. All specialty societies affiliated with the AMB were invited to participate. This first report includes anesthesiology and the 14 surgical specialty societies that accepted the invitation and completed the project during this phase.

Each participating society established a Scientific Committee, generally composed of two members, which independently developed a preliminary catalogue of psychomotor competencies considered essential for undergraduate medical education and assigned the minimum expected competence level for each competency (master the technique, perform independently, or perform under supervision). The principal investigators subsequently standardized terminology, formatting, and wording without modifying the technical content proposed by the Scientific Committees.

### Phase 2: Modified Delphi consensus

The national modified Delphi process was conducted in two anonymous rounds between August and December 2025 and between February and June 2026. Recruitment, questionnaire distribution, and data collection were coordinated centrally by the Brazilian Medical Association using its national database of board-certified specialists from all geographic regions of Brazil. Invitations and questionnaires were distributed electronically by email. Eligible participants were physicians holding specialist certification recognized by the Brazilian Medical Association in one of the participating specialties and listed in the AMB national database. Participation was voluntary. Specialists who completed the first-round questionnaire but did not respond to the second-round email invitation were considered lost to follow-up and were not replaced. No participant was excluded between rounds for reasons other than nonresponse to the second-round invitation.

### Delphi questionnaire

Each questionnaire item presented one psychomotor competency together with the minimum expected competence level previously proposed by the corresponding Scientific Committee. Participants indicated their agreement with each competency-competence level combination using a five-point Likert scale (strongly agree, partially agree, neither agree nor disagree, partially disagree, and strongly disagree).

When different Scientific Committees proposed different competence levels for the same psychomotor competency, each competency-competence level combination was presented separately, allowing independent evaluation of each proposed level.

At the end of the first Delphi round, participants could suggest additional competencies, identify omissions, recommend wording modifications, or report inconsistencies through an open-ended comment field. All comments were reviewed by the principal investigators. Only suggestions originating from participants were considered during questionnaire revision.

### Consensus criteria

Agreement was calculated as the combined proportion of responses classified as strongly agree and partially agree. Competencies achieving ≥75% agreement were considered to have reached consensus. Competencies receiving 60.0–74.9% agreement after the first round were revised according to participant feedback and reassessed during the second round, whereas those receiving <60% agreement were excluded. Additional competencies suggested by participants were incorporated into the second-round questionnaire after review by the Scientific Committees. Only competencies achieving the predefined consensus threshold after the second round were incorporated into the final catalogue.

### Final catalogue and data analysis

The Delphi process evaluated 343 preliminary competency statements. After exclusion of 70 competencies that failed to achieve consensus and incorporation of 90 additional competencies that reached consensus after participant suggestions, the final catalogue comprised 363 specialty-specific psychomotor competencies (Table 1). After harmonization and removal of duplicate competencies shared across specialties, the catalogue contained 244 unique psychomotor competencies. Participant characteristics and competency distributions were analyzed descriptively using absolute numbers and percentages. The final distribution of competencies according to expected competence level is presented in Table 2, and the complete specialty-specific catalogues are provided in Supplementary Tables S1–S15.

**Table 1:** Delphi process and final specialty-specific psychomotor competencies by participating specialty.

Participating specialty	Competencies evaluated in Round 1, n	Excluded for lack of consensus, n	Competencies achieving consensus in Round 1, n	Competencies added after expert suggestions, n	Added competencies achieving consensus, n	Final competencies achieving consensus, n
Anesthesiology	29	5	24	15	14	38
Head and Neck Surgery	16	2	14	5	4	18
Digestive Surgery	29	14	15	2	2	17
Cardiac Surgery	36	15	21	3	2	23
Trauma Surgery	27	2	25	10	8	33
General Surgery	28	4	24	8	6	30
Surgical Oncology	32	7	25	2	2	27
Pediatric Surgery	20	0	20	15	11	31
Plastic Surgery	14	1	13	2	2	15
Thoracic Surgery	23	5	18	2	2	20
Vascular Surgery	17	1	16	10	10	26
Coloproctology	15	2	13	6	6	19
Neurosurgery	38	12	26	2	2	28
Hand Surgery	15	0	15	6	5	20
Urology	4	0	4	15	14	18
**Overall**	**343**	**70**	**273**	**103**	**90**	**363**

Round 1 competencies were independently evaluated by the specialty-specific Scientific Committees using a five-point Likert scale. Competencies achieving the predefined consensus threshold (≥75% combined agreement for “strongly agree” and “partially agree”) were retained. Competencies that did not reach consensus were excluded. Additional competency statements suggested by participants during the first Delphi round were reviewed by the Scientific Committees and, when considered relevant, were incorporated into the second Delphi round. Only additional competencies achieving the predefined consensus threshold were included in the final catalogue.

**Table 2:** Distribution of specialty-specific psychomotor competencies according to the expected competence level at graduation.

Specialty	Total validated competency statements	Master the technique	Perform independently	Perform under supervision
Anesthesiology	38	2	32	4
General Surgery	30	2	24	4
Digestive Surgery	17	2	11	4
Cardiac Surgery	23	2	19	2
Head and Neck Surgery	18	2	12	4
Hand Surgery	20	2	18	0
Neurosurgery	28	2	24	2
Surgical Oncology	27	2	18	7
Pediatric Surgery	31	2	22	7
Plastic Surgery	15	4	9	2
Thoracic Surgery	20	3	8	9
Trauma Surgery	33	3	26	4
Urology	18	2	15	1
Vascular Surgery	26	2	21	3
Coloproctology	19	2	15	2
Specialty-specific subtotal	363	34	274	55
Unique psychomotor competencies	244	5	—	—

Note: Validated psychomotor competencies represent technical motor skills that achieved the predefined consensus threshold in the modified Delphi process (≥75% agreement). The specialty-specific subtotal includes identical or technically equivalent psychomotor competencies that were independently validated by different surgical specialties. After harmonization and removal of duplicate competencies across specialties, the final National Consensus Catalog comprised 244 unique psychomotor competencies.

## RESULTS

### Characteristics of the expert panel

A total of 802 board-certified specialists participated in the first Delphi round, and 622 completed the second round, corresponding to an overall attrition rate of 22.4% (180/802). Attrition resulted exclusively from nonresponse to the second-round questionnaire, and nonrespondents were not replaced. Among participants with available geographic information, 184 (54.6%) were from the Southeast region, 72 (21.4%) from the South, 41 (12.2%) from the Northeast, 23 (6.8%) from the Central-West, and 17 (5.0%) from the North.

### Delphi consensus process

The preliminary catalogues comprised 343 specialty-specific psychomotor competency statements. Following the first Delphi round, 273 competencies (79.6%) achieved the predefined consensus threshold, whereas 70 (20.4%) were excluded because they did not reach consensus. During the first round, participants proposed 103 additional psychomotor competencies, of which 90 (87.4%) achieved consensus after evaluation in the second Delphi round. Consequently, the final national catalogue comprised 363 specialty-specific psychomotor competencies.

### Final national consensus catalogue

After harmonization and removal of competencies duplicated across specialties, the final catalogue contained 244 unique psychomotor competencies. Most competencies were classified at the expected competence level of perform independently (n=274), followed by perform under supervision (n=55) and master the technique (n=34) (Table 2). The distribution of competencies varied among specialties, ranging from 15 competencies in Plastic Surgery to 38 in Anesthesiology (Table 1). The complete specialty-specific catalogues are provided in Supplementary Tables S1–S15.

## DISCUSSION

The present study established the first national consensus on essential psychomotor competencies and their corresponding minimum expected competence levels for graduating medical students in anesthesiology and 14 surgical specialties in Brazil. The final catalogue comprised 363 specialty-specific psychomotor competencies, corresponding to 244 unique psychomotor competencies after harmonization, with most competencies classified as perform independently (n=274), followed by perform under supervision (n=55) and master the technique (n=34).

The proposed catalogue establishes a standardized national reference for the minimum psychomotor competencies expected at the completion of undergraduate medical education while preserving the educational perspectives of each participating specialty. By defining both the competencies and the expected level of performance for each procedure, the catalogue provides a practical framework to support competency-based curriculum development, procedural skills training, and competency assessment in Brazilian medical schools.

The findings are consistent with the principles of competency-based medical education, in which clearly defined competencies and explicit performance expectations constitute the foundation for curriculum planning, teaching, assessment, and the progressive development of clinical autonomy^16,17^. Contemporary competency frameworks emphasize the level of supervision required to perform procedures safely in clinical practice and the progressive development of learner autonomy rather than the mere execution of technical tasks^16,17,18^. In this context, nationally agreed expected competence levels for psychomotor competencies provide a common educational standard that may reduce variability in undergraduate procedural training while supporting more consistent curriculum design, assessment, and alignment with broader competency-based educational goals^19,20^. A distinctive contribution of this study is the integration of independently developed specialty-specific catalogues into a single national catalogue while preserving the educational priorities of each participating specialty. This approach acknowledges that procedural expectations vary across specialties while demonstrating that national consensus can be achieved regarding the minimum psychomotor competencies expected of graduating medical students. The resulting catalogue balances national standardization with specialty-specific expertise, providing a common framework without compromising the educational identity of individual disciplines.

The proposed catalogue also provides a practical framework for competency-based implementation in undergraduate medical education. Clearly defined psychomotor competencies and expected competence levels may support curriculum planning, simulation-based education, workplace learning, and structured assessment, facilitating alignment between teaching activities and expected learning outcomes^21,22,23,24^. Simulation and structured procedural training have been shown to support technical skill acquisition among medical students, including in Brazilian undergraduate settings^22,23,24,25^. By providing explicit performance expectations, the catalogue may contribute to greater consistency in procedural skills training across medical schools and support objective assessment of psychomotor competence.

### Strengths

This study has several strengths. It represents the first nationally coordinated initiative to establish consensus on psychomotor competencies expected of Brazilian medical graduates across multiple specialties using a structured modified Delphi methodology. The participation of Scientific Committees from national specialty societies and a large panel of board-certified specialists provided broad professional representation and strengthened the credibility of the consensus process.

### Limitations

This study also has limitations. First, this manuscript represents Part I of the National Consensus Catalogue and includes only anesthesiology and the surgical specialty societies that accepted the invitation to participate in this phase of the project. Consequently, the proposed catalogue should not be interpreted as representing all medical specialties affiliated with the Brazilian Medical Association. Second, participation in both the Scientific Committees and the Delphi panel was voluntary, introducing the possibility of selection bias. Third, although the modified Delphi method is well established for achieving expert consensus, it provides evidence of content validity through structured consensus rather than psychometric validation or direct evidence of educational effectiveness. Finally, this study did not evaluate the implementation of the catalogue or its impact on learner performance, curriculum development, or patient care outcomes, which should be addressed in future research.

## CONCLUSIONS

This national modified Delphi study developed a consensus catalogue of essential psychomotor competencies and established the corresponding expected competence levels for graduating medical students in anesthesiology and 14 surgical specialties in Brazil. The final catalogue comprised 244 unique psychomotor competencies derived from 363 specialty-specific psychomotor competencies that achieved the predefined Delphi consensus threshold. These findings provide a national consensus-based reference for defining psychomotor competency expectations in undergraduate medical education. Further studies are needed to evaluate the implementation, measurement properties, educational impact, and applicability of the catalogue in different institutional and healthcare settings.

## Data Availability

The datasets generated and analyzed during the current study are available from the corresponding author upon reasonable request.

## References

[1] Frank JR, Snell LS, Ten Cate O, Holmboe ES, Carraccio C, Swing SR (2010). Competency-based medical education: theory to practice. Med Teach.

[2] Englander R, Frank JR, Carraccio C, Sherbino J, Ross S, Snell L (2017). Toward a shared language for competency-based medical education. Med Teach.

[3] Association of American Medical Colleges (2024). Foundational Competencies for Undergraduate Medical Education [Internet].

[4] Urushibara-Miyachi Y, Matsushita K, Nishigori H (2024). Development of a competency-based national model core curriculum in Japan: a case study. Med Teach.

[5] Jennebach J, Özkaya J, Giesecke S, Meißner N, Seidel M, Wyszynski V (2026). National Competence Based Catalogue of Learning Objectives for Undergraduate Medical Education (NKLM) – process description over the last 15 years. GMS J Med Educ.

[6] Michaud PA, Jucker-Kupper P (2017). PROFILES: principal objectives and framework for integrated learning and education in Switzerland.

[7] ten Cate O (2005). Entrustability of professional activities and competency-based training. Med Educ.

[8] Brasil. Ministério da Educação. Conselho Nacional de Educação. Câmara de Educação Superior. Resolução no 3, de 20 de junho de 2014 (2014). Institui as Diretrizes Curriculares Nacionais do Curso de Graduação em Medicina e dá outras providências.

[9] Boursicot K, Etheridge L, Setna Z, Sturrock A, Ker J, Smee S (2011). Performance in assessment: consensus statement and recommendations from the Ottawa conference. Med Teach.

[10] Keeney S, Hasson F, McKenna HP (2011). The Delphi Technique in Nursing and Health Research.

[11] Niederberger M, Schifano J, Deckert S, Hirt J, Homberg A, Köberich S (2024). Delphi studies in social and health sciences: recommendations for an interdisciplinary standardized reporting (DELPHISTAR). Results of a Delphi study. PLoS One.

[12] Diamond IR, Grant RC, Feldman BM, Pencharz PB, Ling SC, Moore AM (2014). Defining consensus: a systematic review recommends methodologic criteria for reporting of Delphi studies. J Clin Epidemiol.

[13] Gattrell WT, Logullo P, van Zuuren EJ, Price A, Hughes EL, Blazey P (2024). ACCORD (ACcurate COnsensus Reporting Document): a reporting guideline for consensus methods in biomedicine developed via a modified Delphi. PLoS Med.

[14] Tallo FS, Abib SCV, Baitello AL, Lopes RD (2014). An evaluation of the professional, social and demographic profile and quality of life of physicians working at the prehospital emergency medical system (SAMU) in Brazil. Clinics (Sao Paulo).

[15] Nasa P, Jain R, Juneja D (2021). Delphi methodology in healthcare research: how to decide its appropriateness. World J Methodol.

[16] Ten Cate O, Carraccio C, Damodaran A, Gofton W, Hamstra SJ, Hart D (2021). Entrustment decision making: extending Miller’s Pyramid. Acad Med.

[17] Touchie C, ten Cate O (2016). The promise, perils, problems and progress of competency-based medical education. Med Educ.

[18] Sawatsky AP, O’Brien BC, Hafferty FW (2022). Autonomy and developing physicians: reimagining supervision using self-determination theory. Med Educ.

[19] Holmboe ES, Sherbino J, Englander R, Snell L, Frank JR, ICBME Collaborators (2017). A call to action: the controversy of and rationale for competency-based medical education. Med Teach.

[20] Gruppen LD, Mangrulkar RS, Kolars JC (2012). The promise of competency-based education in the health professions for improving global health. Hum Resour Health.

[21] Ericsson KA (2008). Deliberate practice and acquisition of expert performance: a general overview. Acad Emerg Med.

[22] Issenberg SB, McGaghie WC, Petrusa ER, Lee Gordon D, Scalese RJ (2005). Features and uses of high-fidelity medical simulations that lead to effective learning: a BEME systematic review. Med Teach.

[23] Cook DA, Hatala R, Brydges R, Zendejas B, Szostek JH, Wang AT (2011). Technology-enhanced simulation for health professions education: a systematic review and meta-analysis. JAMA.

[24] Elendu C, Amaechi DC, Okatta AU, Amaechi EC, Elendu TC, Ezeh CP (2024). The impact of simulation-based training in medical education: a review. Medicine (Baltimore).

[25] Tallo FS, Vendrame LS, Baitello AL (2020). Comparison of three methods for teaching mechanical ventilation in an emergency setting to sixth-year medical students: a randomized trial. Rev Assoc Med Bras (1992).

